# Supplementary material for: A trend analysis of Black American women with cardiovascular disease and chronic medical conditions, sociodemographic factors from NHANES From 2011 to 2020
Source: PLOS Glob Public Health. 2023 Dec 18;3(12):e0002521. doi: 10.1371/journal.pgph.0002521 (PMC10727433; doi:10.1371/journal.pgph.0002521)
Supplement: Attachment — Submitted filename: Reviewer of CVD.docx [file pgph.0002521.s001.docx]

Reviewer #1: I do not think this paper has the required quality. The p value for trends are significant, but they are not well discussed, so their reasons are not known. Although the sample is called African American women, some are born outside the US, so this is probably Black not African American. Most national studies collect data on race (Black or African American) not ethnicity. So, I think this paper should be on Black women because it may have foreign born Black people too. Other than this, title is only on trends, but a major part of the paper is on correlates and risk factors as well. So, in fact, there are two main aims, not one aim.

Author Reponses:

Thanks for your valuable comment and we agree with you. According to your suggestion (Black American), we check the data and updated in the manuscript. And we rewrite the discussion of significant value. Hope this version will be more under stable.

Reviewer #2: Comment to Author
Abstract: Results in abstract are written very poor. for example; from 2nd line to 5th line''
what is the meaning of significant trend in CVD, author should have written some results, should have given some number like the prevalence of CVD has increased/decreased from XXX in 2009 to xxx in 2020.
from 3rd to 5th line: What does author wanted to say with this line?
"These women were more likely to report an education level less than 9th grade, their marital status as widowed/divorced/separated, and having a history of hysterectomy, diabetes, asthma, overweight, arthritis, and or depression."
Please rewrite a meaningful results.

Author Reponses:

Thanks for your comment. We rewrite this section with highlighted line 36-41. Hope this version will be more under stable.

“People with CVD problems 20 years of age and older were included in the analyses. Black American women experienced a considerable prevalence of CVD from 2011 to 2020. These women were more likely to report having completed no more schooling than the ninth grade, being widowed/divorced/separated, and having undergone a hysterectomy as well as having a history of diabetes, asthma, obesity, arthritis, and depression. Black American women with CVD had a 3.8-fold increased risk of diabetes and a 5.6-fold increased risk of arthritis.”

Introduction: 1. There are very few arguments has been given in introduction section about the topic.
2. What is the rationale behind the study?

Author Reponses:

Thanks for your comment. We rewrite this section with highlighted. Hope this version will be more under stable.

“Compared to other racial and ethnic groups, Black American women have been found to have higher rates of CVD, other non-communicable chronic medical problems, and worse outcomes (Ebong et al., 2020). In order to address these discrepancies, it can be useful to understand the trends in CVD and other chronic illnesses among Black American women (Carnethon et al. 2017). In the United States, CVD is the primary cause of mortality for women (Carnethon et al. 2017). Non-communicable chronic illnesses including diabetes, asthma, and arthritis all significantly increase the burden of illness (Ebong et al., 2020). Public health professionals can better allocate resources for their management and prevention by looking at trends in these disorders. Chronic diseases like CVD, diabetes, and arthritis can have serious long-term health effects that can result in disability, a decline in quality of life, and a rise in medical expenses (Maresova et al., 2020). The long-term health outcomes for Black American women can be improved through public health policies and interventions (Maresova et al., 2020), which are informed by trends in these conditions.

Previous studies have indicated that Black American women are more likely than other demographic groups to experience cardiovascular disease and other chronic medical disorders (Ebong et al., 2020) (Maresova et al., 2020). Socioeconomic position, access to healthcare, genetics, and lifestyle elements including nutrition and physical activity levels all may play a role in this discrepancy (Pampel et al., 2010).

According to DeSalvo et al. (2016), research on socioeconomic determinants of health and how they affect health outcomes is desperately needed, especially for populations who are more likely to experience health disparities. Moreover, looking into trends in sociodemographic factors over the previous ten years may help in identifying areas for improvement and informing targeted interventions to reduce health disparities given the ongoing COVID-19 pandemic and its impact on health outcomes, including cardiovascular disease and chronic medical conditions (Kwok et al., 2021). Overall, the importance of this study issue may be seen in its potential to shed light on the patterns of CVD and other chronic medical conditions among Black American women, which can help guide plans for enhancing health outcomes and eradicating health disparities in this community.”

Methodology
1. Authors of this manuscript did not mentioned about data source. Methodology section directly started with study variables and directly jump to statistical analysis, which is not the proper method to write an methodology section of any paper.
Authors of this paper should have given a paragraph about th data source. Author need to write some information about data like, how data was collected and sampling design or where data is available.

Author Reponses:

Thanks for your comment. We rewrite the methodology section according to your suggestion line 106-168. Hope this version will be more under stable.

“***Study Population and Design***

The National Health and Nutrition Examination Survey (NHANES), an ongoing cross-sectional study that aims to estimate the prevalence and distribution of various diseases and risk factors in the United States, is managed by the Centers for Disease Control and Prevention's National Center for Health Statistics. NHANES oversamples a variety of distinct demographic groups, including those who are Hispanic, non-Hispanic Black, non-Hispanic Asian, older adults, and low-income persons, in order to offer appropriate samples for subgroup analysis and more precise variance estimates. Only data from Black American individuals in the NHANES 2011-2020 survey were used in our analysis. Age, family income to poverty, country of birth, education level, marital status, vigorous work activity, and moderate work activity were among the demographic details that participants self-reported. A doctor confirmed that she had undergone a hysterectomy, diabetes, asthma, obesity, arthritis, cancer, and depression. All participants provided written informed consent for the study, which received approval from the NCHS institutional review board (King & Xiang, 2017).

***Data Collection and Measures***

The study team interviewed participants at their homes to gather data on their age, family income to poverty level, country of birth, degree of education, marital status, and level of vigorous and moderate work activity. Additionally, during a consultation held at the examination center, doctors confirmed the existence of chronic medical conditions such as hysterectomy, diabetes, asthma, overweight, arthritis, cancer, and depression.

***Measures (Dependent Variable)***

Four NHANES cycles—2011–2012, 2013–2014, 2015–2016, and 2017–2020—were used to collect the data we used. The population of Black American women with CVD was the dependent variable in our study. Any affirmative response connected to any of the following variables was used to define CVD: MCQ160a (Ever told you had congestive heart failure), MCQ160b (Ever told you had congestive heart failure), MCQ160c (Ever told you had coronary heart disease), MCQ160d (Ever told you had angina/angina pectoris), MCQ160e (Ever told you had a heart attack), and MCQ160f (Ever told you had a stroke).

***Other measures (Independent Variables)***

The study used a range of independent variables to examine their relationships to chronic illnesses like CVD and others in Black American women. Age, family income to poverty ratio, place of birth, level of education, married status, level of vigorous and moderate work activity, hysterectomy, diabetes, asthma, overweight, arthritis, cancer, and depression were among these factors. The RIDRETH1 and RIAGENDR variables were utilized to represent race and gender, respectively. RIDAGEYR was used to calculate age, INDFMPIR to calculate family income, DMDEDUC2 to calculate education, and DMDMARTL to calculate marital status. DMDBORN4 was used to establish the birthplace, PAQ605 was used to measure strenuous work activity, and PAQ620 was used to determine moderate work activity. Diabetes was detected using DIQ010, hysterectomy by RHD280, asthma by MCQ010, obesity by MCQ080, stroke by MCQ160f, cancer by MCQ220, and depression by the Patient Health Questionnaire, a nine-item depression screening tool that incorporates DSM-IV diagnostic criteria. Scores over 5 on the measure, which employs a point system based on responses to each item, signify a depressed condition. (Kroenke & Spitzer, 2002; Kroenke et al., 2001, Spitzer et al., 1999).

***Statistical Analysis:***

Oversampling, stratification, and clustering are some of the complicated survey design techniques used by the National Health and Nutrition Examination Survey (NHANES). Each participant is given a weight to account for varying sampling likelihood and nonresponse. In this study, for each two-year survey cycle from 2011 to 2020, we computed the prevalence (%) of CVD among Black Americans. And age, ratio of family income to poverty, country of birth, education level, marital status, vigorous work activity, moderate work activity, hysterectomy, diabetes, asthma, overweight, arthritis, cancer, and depression stratified by CVD prevalence, using survey methodologies for all analyses. We addressed the complex survey design, including oversampling, survey nonresponse, and poststratification, using the 2-year weights computed using the NCHS (National Center for Health Statistics) technique. SURVEYFREQ and SURVEYREG were two methods we used to analyze the data (King & Xiang, 2017). We conducted a 2-analysis using SURVEYFREQ to see if there was a difference. The SURVEYREG method was employed for the regression analysis. To examine linear trends over survey cycles and calculate P values for trends, we used weighted logistic regression. In the regression models, the survey cycle was treated as a continuous variable. Missing data were not included in the study. The programming language R was employed to carry out the investigation. P<0.05 (two-sided) was used to determine statistical significance (King & Xiang, 2017).”

2. what is meaning of this line in statistical analysis section: "We used weighted logistic regression to examine linear trends (crude) across survey cycles to determine P values for trends." I could not get it that logistic regression was used to determine p values for trends. what is the meaning of this line?

Author Reponses:

Thanks for your comment.

This line indicates that weighted logistic regression was employed by the researchers to look at linear trends in the prevalence of CVD among Black American women over time. The logistic regression models were not modified for additional factors that could affect the link between time and the prevalence of CVDs, which is what is meant by the "crude" aspect. We were able to assess whether there was a statistically significant change in the prevalence of CVD among Black American women throughout the course of the survey cycles by analyzing these trends. The obtained P values provided statistical significance levels for any observed trends, enabling to make inferences regarding the association between time and the prevalence of CVD.

Results
line 133 to 135 "The prevalence of those with education level less than 9th grade". Does this line means that percentage of individuals with education level less than 9th grade....?
Author may want to write the prevalence of CVD among those with educational level 9th grade.....
Like this there are many results are not written very carefully whuile interpreting and creating confusion. All the results of this paper also written very poorly. I would suggest please rewrite again the result section.

Author Reponses:

Thanks for your comment. We rewrite the result section line 173-208. Hope this version will be more under stable.

“The table 1 shows the prevalence percentages of CVD and numerous other variables among Black American women over the course of various years. The total prevalence of CVD rose from 7.92% in 2013–2014 to 10.9% in 2015–2016 before dipping slightly to 10.2% in 2017–2020 (P for trend = 0.03). The participants' mean age grew from 43.22 to 46.27 years between 2011 and 2020 (P for trend = 0.053). Over the course of the study, there was no discernible trend in the proportion of family income to poverty (P for trend = 0.435). Although the prevalence of CVD among Americans born between 2011 and 2012 and 2017 and 2020 increased, this trend was not statistically significant (P for trend = 0.543). According to education level, CVD prevalence was highest among those with less than a ninth-grade education (41%) and lowest among those with a college degree or higher (7%) in the same year. Statistics showed that there were disparities in the ninth-grade education of education (P = 0.0004). The prevalence of CVD varied significantly depending on marital status as well. In the years between 2017 and 2020, individuals who were widowed, divorced, or separated had the highest prevalence of CVD (32%) while those who were married or cohabiting had the lowest prevalence (9%). There were statistically significant variations in the groups according to marital status (P = 0.014). Throughout the study period, there were statistically significant differences between groups in the prevalence of hysterectomy, diabetes, asthma, arthritis, and depression. For instance, the prevalence of hysterectomy rose from 27% to 25% between 2011 and 2020 (P 0.001), while the prevalence of asthma fell from 15% to 13% between 2011 and 2020 (P = 0.004). With the highest prevalence in 2015-2016 (14%) and the lowest prevalence in 2013-2014 (6%), the prevalence of overweight also varied throughout the course of the study period and revealed a statistically significant difference between groups (P = 0.044). The prevalence of cancer increased non-significantly (P = 0.054) from 25% in 2011–2012 to 33% in 2017–2020 (Figure:1).

Table 2 displays the relationships between key research variables and CVD in Black American women from 2011 to 2020. The following study factors were statistically significant: Being born in the US: Compared to women who were not born in the US, those who were born in the US had a 3.017 times higher risk of developing CVD. Education level: Compared to women with college degrees or above, women with less than a ninth-grade education had 0.213 times reduced odds of having CVD. Relationship status: Compared to women who were married or cohabiting, women who were widowed, divorced, or separated had 2.772 times higher odds of having CVD. Hysterectomy: Women who underwent a hysterectomy had a 2.879-times-higher risk of developing cardiovascular disease (CVD) than women who did not. Women who had diabetes, asthma, obesity, arthritis, and depression had increased probabilities of developing CVD than women who did not have these illnesses. The odds ratios were, correspondingly, 3.813, 1.918, 1.533, 5.575, and 1.718.”
