# Supplementary material for: A trend analysis of Black American women with cardiovascular disease and chronic medical conditions, sociodemographic factors from NHANES From 2011 to 2020
Source: PLOS Glob Public Health. 2023 Dec 18;3(12):e0002521. doi: 10.1371/journal.pgph.0002521 (PMC10727433; doi:10.1371/journal.pgph.0002521)
Supplement: Attachment — Submitted filename: Reviewer_Ripon (3).docx [file pgph.0002521.s002.docx]

Reviewer #3: This study uses NHANES data to look at how CVD burdens in Black women have changed between 2021 and 2020. The findings are meaningful and have potential clinical and policy implications.

Authors Responses:

Thanks for your valuable comment.

Major issues:
1. Results: the authors started with Table 2. I cannot seem to find Table 1. Not sure if it was a typo or that they left out Table 1 by accident.

Authors Responses:

Thanks for your comment. We corrected it.

2. The authors should briefly mention by the other release cycles include data from two years, but the 2017-2020 included data for 4 years. Providing this kind of technical notes and details are important for contexts.

Authors Responses:

Due to the coronavirus disease 2019 (COVID-19) pandemic, the NHANES program stopped conducting fieldwork in March 2020. As a result, data collection for the NHANES 2019-2020 cycle was not finished, and the data that was gathered were not representative of the entire country. Because of this, data from the NHANES 2017–2018 cycle were joined with data from the 2019–March 2020 cycle to provide a nationally representative sample of pre-pandemic NHANES 2017–2020 data. That’s why authors included 4 years data for the 2017-2020 release cycle for more updated information (*NHANES Questionnaires, Datasets, and Related Documentation*, 2023.)

3. The missing data issues are complicated NHANES. Did the authors conduct any missing data analysis? Were the data missing at random? This is a major issue and needs to be addressed.
Authors Responses:

Participants with missing data were excluded from this study for maintaining accuracy, removing biases as well as enhancing the statistical power of the study. Another study dealing with NHANES data excluded the participants with missing data from the study during statistical analysis for the same reason (Moonesinghe et al., 2019).

4. Line 162-163, the authors stated that they "conducted a 2-analysis using XXXX." Is this a typo? Not sure what is a "2-analysis." Also, to see there was a difference of what? Clarifications and more details are needed.

Authors Responses:

Thanks for your valuable comment. For more addressable, we removed it.

“We conducted an analysis using SURVEYFREQ to determine the prevalence of various responses and identify patterns or trends within the data.”

5. Figure 2: the title (both on top in red text and italicized texts on the bottom) is awkward phrased. Also, the y-axis in each plot did not start at 0, which skewed the actual magnitude of changes, i.e., making changes look bigger than they actually are. Also, 95% confidence intervals should be included to indicate whether the changes were significant.

Authors Responses:

Thanks for your valuable comment. Figure 1 can be replaceable for Table 1. For this reason, authors decided to remove the figure 1.

6. Line 549: i assume the dot was a typo? Also, using on asterisk for statistical significance and two asterisks for reference group was confusing. I suggest they pick a different symbol for the reference group notation.

Authors Responses:

Thanks for your valuable comment. For more addressable, we edited it.

Minor issues:

1. I don't think it is necessary to include the actual variable names in the descriptions of the measurement. In fact i have never seen that done in studies using NHANES or any other secondary datasets. It is unnecessary, and might even be confusing and distracting to readers not familiar with NHANES datasets. I think they need to be removed.

Authors Responses:

Thanks for your valuable comment.

With respecting your thinking and considering further work on this, we decided to remain it. Also, in this perspective second reviewer agreed with us.

2. Line 166, need to provide a citation for the R program. Line 160, need to include a citation for the NCHS policy and weighting instructions.

Authors Responses:

Thanks for your valuable comment. For more addressable, we edited it.

1. Bilder, Christopher R, and Thomas M Loughin. 2014. Analysis of Categorical Data with r. CRC Press.
2. CDC. 2018. “NHANES Web Tutorial Frequently Asked Questions (FAQs).” <https://www.cdc.gov/nchs/tutorials/NHANES/FAQs.htm>.
3. CDC,NCHS. 2018. “National Health and Nutrition Examination Survey Data.” <https://wwwn.cdc.gov/nchs/nhanes/>.
4. Dhana, A. 2018. “Data Science ++.” <https://datascienceplus.com/>.
5. Dobson, A. J., and A. G Barnett. 2008. An Introduction to Generalized Linear Models, Third Edition. Chapman; Hall/CRC.
6. Flegal, Katherine M, Deanna Kruszon-Moran, Margaret D Carroll, Cheryl D Fryar, and Cynthia L Ogden. 2016. “Trends in Obesity Among Adults in the United States, 2005 to 2014.” Jama 315 (21): 2284–91.
7. Heeringa, Steven G, Brady T West, and Patricia A Berglund. 2017. Applied Survey Data Analysis. Chapman; Hall/CRC.
8. Lumley, Thomas. 2011. Complex Surveys: A Guide to Analysis Using r. Vol. 565. John Wiley & Sons.
9. Susmann, Herb. 2016. RNHANES: Facilitates Analysis of CDC NHANES Data. [https://CRAN.R-project.org/package=RNHANES](https://cran.r-project.org/package=RNHANES).
10. Vittinghoff, Eric, David V Glidden, Stephen C Shiboski, and Charles E McCulloch. 2011. Regression Methods in Biostatistics: Linear, Logistic, Survival, and Repeated Measures Models. Springer Science & Business Media.
11. *NHANES Questionnaires, Datasets, and Related Documentation*. (n.d.). Retrieved July 31, 2023, from https://wwwn.cdc.gov/nchs/nhanes/continuousnhanes/default.aspx?Cycle=2017-2020
12. Moonesinghe, R., Yang, Q., Zhang, Z., & Khoury, M. J. (2019). Prevalence and Cardiovascular Health Impact of Family History of Premature Heart Disease in the United States: Analysis of the National Health and Nutrition Examination Survey, 2007–2014. *Journal of the American Heart Association*, *8*(14), 2007–2014. https://doi.org/10.1161/JAHA.119.012364
